# Comparison between supine and prone patient setup for lumbosacral spinal stereotactic body radiosurgery with CyberKnife

**DOI:** 10.3389/fonc.2023.959447

**Published:** 2023-04-03

**Authors:** Jun Li, Xianghui Kong, Cheng cheng, Gong Wang, Hongqing Zhuang, Ruijie Yang

**Affiliations:** ^1^ Department of Radiation Oncology, Peking University Third Hospital, Beijing, China; ^2^ School of Radiation Medicine and Protection, Soochow University, Suzhou, China; ^3^ Collaborative Innovation Center of Radiological Medicine of Jiangsu Higher Education Institutions, Suzhou, China

**Keywords:** lumbosacral spinal tumor, supine position, prone position, xsight spine prone tracking, CyberKnife

## Abstract

**Objective:**

The aim of this study is to analyze which tracking modality is more suitable for stereotactic body radiosurgery of lumbosacral spinal tumors by comparing prone and supine patient treatment setup.

**Methods:**

Eighteen patients with lumbosacral spinal tumors were selected. CT simulation was performed in the supine position (fixed with a vacuum cushion) and prone position (fixed with a thermoplastic mask and prone plate), respectively. The plans in the supine and prone positions were designed using the xsight spine tracking (XST) and xsight spine prone tracking (XSPT) modalities, respectively. The dose-volume histogram (DVH) parameters, namely, V_100%_, D_95%_, D_mean_, conformity index (CI), and heterogeneity index (HI) in planning target volume (PTV), as well as D_max_, D_0.1cc_, D_1cc_, and D_5cc_ in the cauda equina and bowel were recorded. The supine plans were simulation plans and were not used for treatment, which were only used to record the alignment errors. The spinal tracking correction errors (alignment error) and correlation errors of the synchrony respiratory model in the prone position were recorded during the treatment. After treatment, the simulation plan of the supine position was implemented and the spinal tracking correction errors were recorded. The parameters of correction error and DVH parameters for the two positions were analyzed using the paired *t*-test to compare the difference in positioning accuracy and dose distribution. In addition, the correlation errors of the synchrony respiratory model in the prone position were analyzed to evaluate the prediction accuracy of the synchrony model.

**Results:**

For patient setup, the correction error of the supine position in interior/posterior was (0.18 ± 0.16) mm and the prone position was (0.31 ± 0.26) mm (*P*< 0.05). The correction error of the supine position in inferior/superior was (0.27 ± 0.24) mm, and the prone position was (0.5 ± 0.4) mm (*P<* 0.05). The average correlation errors of the synchrony model for left/right, inferior/superior, and anterior/posterior in the prone position were (0.21 ± 0.11) mm, (0.41 ± 0.38) mm, and (0.68 ± 0.42) mm, respectively. For the dose distribution, compared with prone plans, the average CI in supine plans was increased by 4.5% (*P*< 0.05). There was no significant difference in HI, PTV V_100%,_ D_95%_, and D_mean_ between the prone and supine plans. Compared with supine plans, average D_1cc_ and D_5cc_ for the cauda equina was significantly decreased by 4.7 and 15.3% in the prone plan (*P<* 0.05). For the bowel, average D_max_, D_0.1cc_, D_1cc_, and D_5cc_ were reduced by 8.0, 7.7, 5.2, and 26.6% in prone plans (*P*< 0.05) compared with supine plans.

**Conclusion:**

Compared with the supine setup, the prone setup combined with XSPT modality for the lumbosacral spinal stereotactic body radiosurgery can spare the bowel and cauda equina of the middle and low dose irradiation, and decrease the number of beams and monitor units.

## 1 Introduction

Stereotactic radiation therapy (SRT) can increase tumor dose and reduce exposure to normal tissue, resulting in higher tumor control rates and lower normal tissue complications (1–3). As a special device for SRT, CyberKnife is increasingly applied in the treatment of spinal tumors in modern radiotherapy (4–6). To ensure the stability of the patient’s position during treatment, CyberKnife is used to treat tumors in the supine position. However, some postoperative spinal patients could not keep the supine position for a long time due to pain. In addition, due to the workspace limitation, the robotic arm suffers from a lack of posterior beams (7). This means that beams have to pass through a length of normal tissue before reaching the target resulting in increased dose to normal tissue when patients were in the supine position. Hence, the prone position treatment mode became a necessary choice. With the system upgrade, the fifth generation CyberKnife (VSI) was able to offer two kinds of spine tracking modalities: xsight spine tracking (XST) and xsight spine prone tracking (XSPT). XST is a spine image registration algorithm which is supported for supine treatments only (8). XSPT combines the spine image registration algorithm with dynamic compensation of respiratory motion, which can realize spinal radiosurgery in the prone position. Although prone position can overcome the above problems, the stability of the position and the reliability of tracking can be reduced due to the influence of respiration (9, 10). Therefore, whether the prone position can be used for spinal tumor treatment needs to be further discussed. However, at present, there are few reports about the use of the XSPT modality. In order to determine which of the two tracking methods, XSP and XSPT, has more advantages or is more suitable for patients with lumbosacral spinal tumors, this study compares the following three aspects:

1. The difference between the target coverage and the dose to organs at risk (OARs) was compared by analyzing the dose distribution of the two methods.
2. The treatment efficiency of the machine was compared by analyzing the beam number, machine monitor unit (MU), and treatment time.
3. The tracking accuracy of the target during treatment was compared by analyzing the positioning errors and the correlation errors of the synchrony model.

## 2 Methods and materials

### 2.1 Patient characteristics

Eighteen patients with lumbosacral spinal tumors were selected, who received CyberKnife treatment in our institute from July 2020 to June 2021. The patient characteristics are shown in **Table 1**. The study was approved by the institutional review board of our institute.

**Table 1:** Patient characteristics.

	Female	Male
Number	8	10
Age	25[22-67]	56[34-65]
Disease	Ewing sarcoma (2) Osteosarcoma (1) Sarcoma (1) Spinal metastases (4)	Osteosarcoma (1) Chordoma (2) Spinal metastases (7)
Surgical status	Surgery (3) No surgery (5)	Surgery (6) No surgery (4)
BMI	21.2[17.2-24.3]	22.6[17.9-25.8]
Site	L3-S1	L2-S1
Number of fractions	3[1-5]	3[1-5]
Prescription dose (Gy)	27[21-35]	30[30-35]
Volume of target (cc)	108.58[38.3-428.67]	153.21[96.2-578.67]
Aperture of IRIS collimators(mm)(median [range])	30[12.5-60]	30[12.5-60]
Number of IRIS collimators (median [range])	3[2-4]	3[2-4]

### 2.2 CT simulation positioning

**Figure 1:**
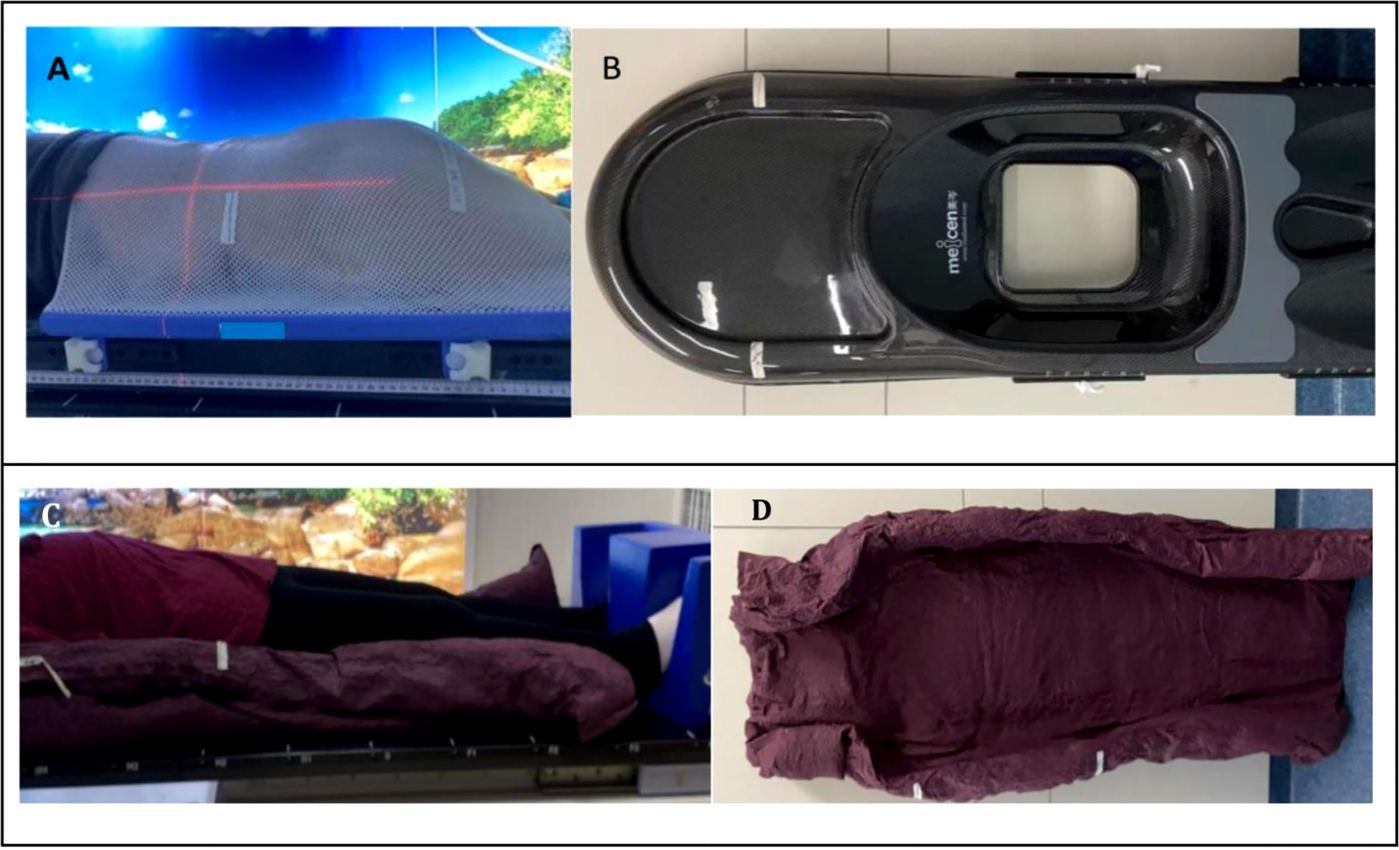
Patient treatment positions and fixation devices, **(A)** treatment setup in the prone position, **(B)** belly board, **(C)** treatment set up in the supine position, **(D)** vacuum cushion.

CT simulation positioning was performed for each patient both in the supine position and prone position. As shown in **Figure 1A**, in the prone position, the patient laid on the carbon fiber belly board and was fixed with the thermoplastic mask. The belly board is a **Figure 1B** central hollow device. It could relieve abdominal compression *via* the support to reduce the motion caused by breathing. In the **Figure 1D** supine position, the patient was fixed using a vacuum cushion with feet support without immobilization devices shown in **Figure 1C**. CT scans were acquired with 1.5 mm slice spacing, and the scanning range should be at least 10 cm from the upper and lower boundaries of the target.

### 2.3 Treatment planning

The target and OARs including the spinal cord and bowel were contoured in the supine and prone data sets, respectively. The planning target volume (PTV) was given as the gross target volume (GTV) plus a margin of 3 mm. In this study, the PTV for the supine and prone positions were generated with the same margin. The planning objectives of OARs followed the RTOG 0631 and AAPM TG101 protocol shown in **Table 2**, and PTV should satisfy V_100%_ larger than 95%. For the plan design parameters, the maximum number of beams and maximum MU per fraction was not larger than 220 and 10000 MU, respectively, and the minimum MU of beams was not less than 5 MU. The same aperture and number of IRIS collimators were used for both XSP and XSPT plans. Sequential optimization was used. The dose was calculated using the RT algorithm. The target tracking modality used XST for the supine position and XSPT for the prone position. Treatment plans were generated using the same aperture and number of collimators both in the supine and prone positions. The initial optimization conditions of the two position plans were consistent, and then the OARs dose and global low-dose volume were further reduced under the premise of meeting the target dose prescription.

**Table 2:** Dose constraints of OARs.

		1 fraction		3 fractions		5 fractions	
OARs	Max critical volume above threshold (cc)	Threshold dose (Gy)	Max point dose (Gy)	Threshold dose (Gy)	Max point dose (Gy)	Threshold dose (Gy)	Max point dose (Gy)
Cauda equina	5	14	16	21.9	24	30	32
Bowel	20	14.3	18.4	24	28.2	25	38

### 2.4 Dosimetric evaluation

The dose-volume histogram (DVH) and delivery efficiency of treatment plans were compared between the supine position and the prone position.

For the target, the dosimetric evaluation indices are as follows:

V_100%_ of PTV: volume of PTV receiving 100% of the prescribed dose

D_95%_ of PTV: the dose 95% of the PTV volume received

D_mean_ of PTV: average dose received by PTV


(1) $$\text{HI}=D_{\text{max}}/D_{\text{pre}}$$

D_max_ and D_pre_ are the maximum dose and the prescribed dose, respectively.


(2) $$\text{CI}=\left(V_{\text{RX}}/V_{\text{T}}\right)\times \left(V_{\text{RX}}/V_{\text{RI}}\right)$$

V_T_ is the volume of PTV, V_RI_ is the volume wrapped by the isodose line of the prescribed dose, and V_RX_ is the volume wrapped by the prescribed dose around PTV

For the OARs, the dose evaluation indices of cauda equina and bowel were as follows:

D_max_: the maximum dose delivered to tissue
D_0.1cc_: the dose delivered to a 0.1-ml volume of tissue
D_1cc_: the dose delivered to a 1-ml volume of tissue
D_5cc_: the dose delivered to a 5-ml volume of tissue

For delivery efficiency, the number of beams, MU, and treatment time were recorded.

### 2.5 Setup evaluation

The plans of the prone position were used for patient treatment. The patient was setup prone on the treatment couch in the same immobilization device that was used during the CT scan. The LED markers were attached to the patient’s thermoplastic mask such that these markers were visible to the camera array for acquisition of respiratory movement signals of patient. At the beginning of treatment, eight pairs of orthogonal X-ray images in the different respiratory phases were taken and registered to the digitally reconstructured radiographs (DRRs) to build the synchrony model for target tracking. During the treatment, orthogonal X-ray images were acquired at an interval of 30–90 s to monitor patient positioning errors and verify the accuracy of synchrony model. In order to analyze the tumor tracking accuracy and prediction accuracy of synchrony model, couch correction errors (inferior/superior, left/right, anterior/posterior, roll, pitch, and yaw), and correlation errors of synchrony model (inferior/superior, left/right, and anterior/posterior) were recorded.

Treatment planning of the supine position as simulation planning was used for evaluating the setup correction errors. The patient was fixed with a vacuum cushion on the treatment couch in the supine position. The orthogonal X-ray images were acquired and registered to the DRRs to obtain the couch correction parameters of six dimensions every 60 s for approximately 20 min.

### 2.6 Statistical analysis

DVH parameters, delivery efficiency, and couch correction errors in the prone and supine positions were compared using the paired two-sided *t*-test. *P* values of< 0.05 were considered to indicate statistical significance. All statistical analyses were performed using the SPSS Statistics 21.0 software program.

## 3 Results

### 3.1 Dosimetric evaluation

**Figure 2:**
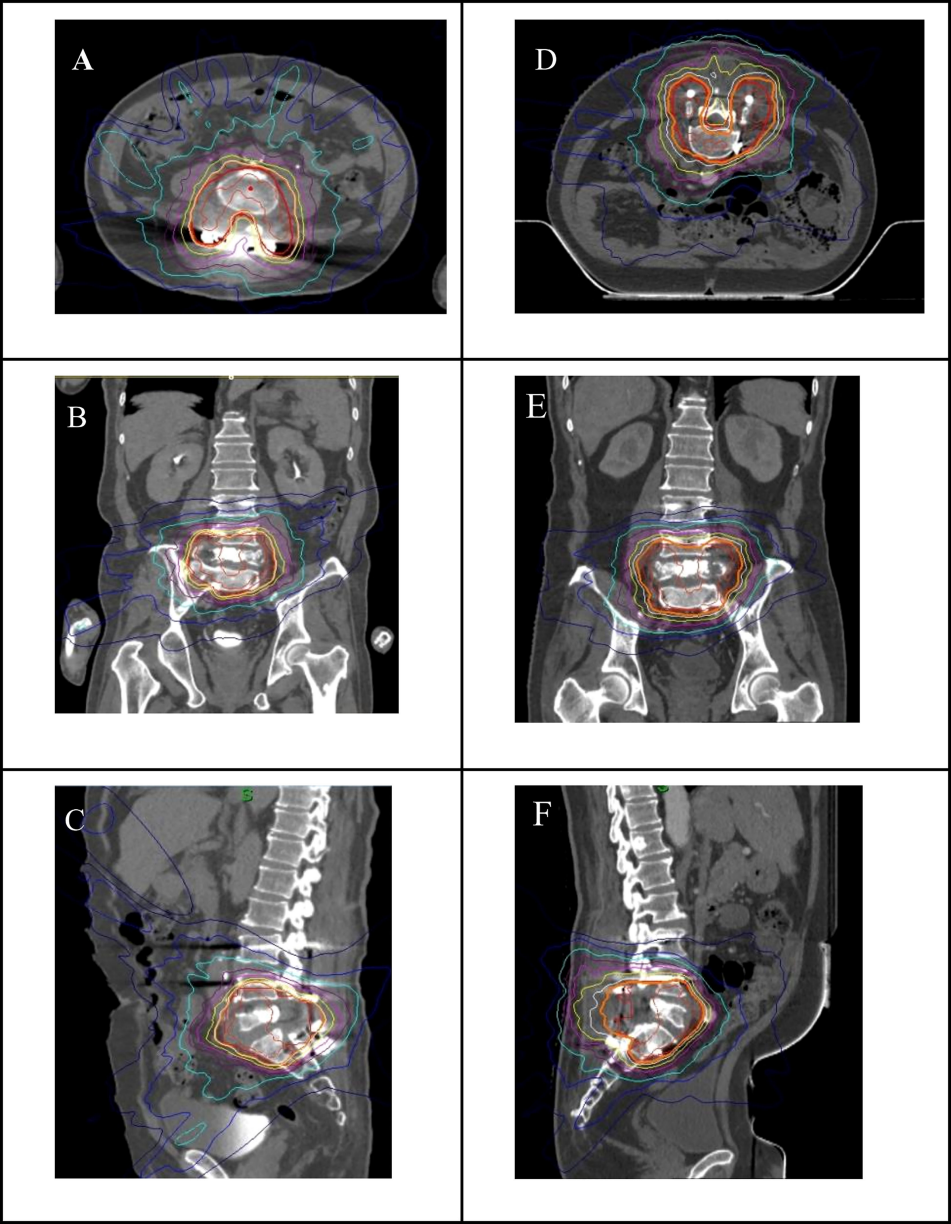
Dose distribution of axial **(A)**, coral **(B)** and sagittal **(C)** CT images in supine position, and dose distribution of axial **(D)**, coronal **(E)** and sagittal **(F)** CT images in prone position.

**Figure 2** showed the dose distribution for lumbar spine lesions between the supine and prone positions. Compared with the supine plan, the volume of the low dose region was more than the prone plan. In order to quantify specifically the effect on the dose differences in the supine and prone positions, the dosimetric parameters were given in **Table 3**. The difference of average PTV V_100%_, D_95%_, D_mean_, and heterogeneity index between the supine plans and prone plans were not significant. Compared with the supine plans, there was better conformity index (CI) for the prone plans. The average CI was 1.34 ± 0.18 and 1.28 ± 0.22 (*p*< 0.001) in supine and prone positions. For the cauda equina, no statistically significant difference was observed in the average D_max_ and D_0.1cc_. However, the average D_1cc_ and D_5cc_ of cauda equina was 4.7 and 15.1% higher in the supine plans than in the prone plans, and the difference was statistically significant. For the bowels, the average D_max,_ D_0.1cc_, D_1cc_, and D_5cc_ were 8.0, 7.7, 5.2, and 26.6% higher in the spine plans, respectively. In addition, compared with the volumes of the PTV and OARs in the two positions, it was found that the volume of the target and the bowel was significantly different, and the PTV and bowel were larger in the supine position than in the prone position.

**Table 3:** Dosimetric differences between supine and prone positions for the patients with lumbosacral spinal tumors.

Dosimetric parameter	Supine	Prone	*p*-value
PTV V_100%_	94.87% ± 0.57%	94.92% ± 0.34%	0.51
PTV D_95%_ (Gy)	30.26 ± 8.96	30.34 ± 9.35	0.27
PTV D_mean_ (Gy)	35.98 ± 9.21	36.17 ± 8.83	0.13
HI	1.48 ± 0.14	1.50 ± 0.11	0.46
CI	1.34 ± 0.18	1.28 ± 0.22	0.04
PTV volume (cc)	178.68 ± 278.67	153.21 ± 324.30	0.02
Cauda equina D_max_ (Gy)	29.26 ± 9.29	28.65 ± 8.95	0.33
Cauda equina D_0.1cc_ (Gy)	28.53 ± 9.45	27.95 ± 8.66	0.21
Cauda equina D_1cc_ (Gy)	26.79 ± 9.66	25.53 ± 8.08	0.04
Cauda equina D_5cc_ (Gy)	21.54 ± 8.28	18.28 ± 7.93	< 0.01
Cauda equina volume (cc)	15.80 ± 1.48	15.61 ± 2.13	0.18
Bowel D_max_ (Gy)	22.12 ± 5.90	20.87 ± 5.52	< 0.01
Bowel D_1cc_ (Gy)	20.34 ± 4.68	19.27 ± 46.7	0.02
Bowel D_0.1cc_ (Gy)	18.41 ± 4.86	17.46 ± 3.83	0.03
Bowel D_5cc_ (Gy)	15.32 ± 5.06	11.24 ± 3.60	< 0.01
Bowel volume (cc)	478.54 ± 287.5	415.54 ± 221.5	< 0.01

### 3.2 Setup and delivery efficiency evaluation

**Figure 3:**
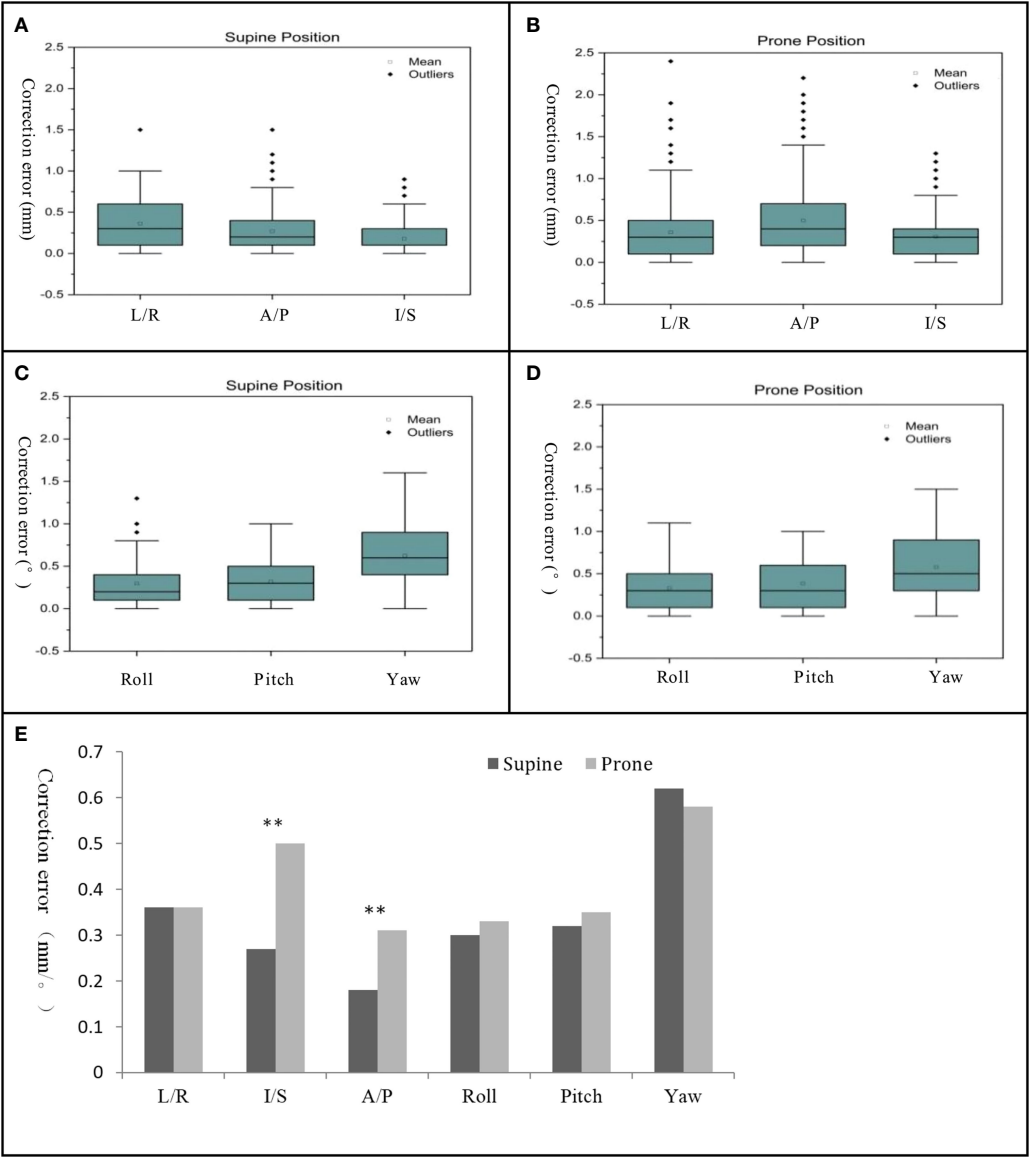
Boxplot of correction error for translation direction in supine position **(A)** and prone position **(B)** and correction error for angle rotation in supine position **(C)** and prone position **(D)** and bar chart of correction errors between supine and prone position **(E)**. "**" denotes P<0.01, there was a statistical difference between supine position and prone position.

As shown in **Table 3**, the correction errors of left/right, inferior/superior and anterior/posterior in the supine position were 0.36 mm ± 0.28 mm, 0.27 mm ± 0.24 mm, and 0.18 mm ± 0.16 mm, respectively. The correction errors of left/right, inferior/superior and anterior/posterior directions in the prone position were 0.36 mm ± 0.32 mm, 0.50 mm ± 0.40 mm, and 0.31 mm ± 0.26 mm, respectively. Compared with the supine position, the prone position had a larger correction error in the direction of inferior/superior and anterior/posterior, and the difference was statistically significant (*P*< 0.05). The correction errors of angle, roll, pitch, and yaw were 0.30° ± 0.26°, 0.32° ± 0.23°, and 0.62° ± 0.34° in the supine position. The corrected errors of prone position were 0.58° ± 0.36°, 0.33° ± 0.27°, and 0.39° ± 0.42°. The correction errors of angle were not significant between the supine and prone positions, and the difference was not statistically significant. The boxplot indicated (**Figure 3**) that the number of outliers in the inferior/superior and anterior/posterior for the prone position is more than that in the supine position. However, the difference of the angle correction error between the supine and prone positions was not significant. For the prone position with XSPT modality, the average correlation error of the synchrony model in left/right, inferior/superior, and anterior/posterior was 0.21 mm ± 0.11 mm, 0.41 mm ± 0.38 mm, and 0.68 mm ± 0.42 mm, respectively, as shown in **Table 4**. The root mean square of average correlation error in three directions was 0.82 mm ± 0.57 mm, and the maximum value is 1.32 mm.

**Table 4:** Correlation error of synchrony model in the prone position.

Left/right	Interior/superior	Anterior/posterior
0.21 mm ± 0.11 mm	0.41 mm ± 0.38 mm	0.68 mm ± 0.42 mm

In the prone plans, the treatment time, MU and beam numbers were 39.42 min ± 9.73 min, 40920 MU ± 2376 MU and 178 ± 3, respectively. For all plans, the prone plans consistently had a lower number of MU and beams. Compared with the supine plans, the number of MU and beams was on average 7.3 and 6.8% lower in prone plans.

## 4 Discussion

Because of the lack of posterior beams of limitations for CyberKnife and the clinical requirements for postoperative patients with spinal tumors, we are prompted to investigate the accuracy of the prone spine treatment mode. Previous studies have shown that the prone position induced larger organ motion compared with the supine (11, 12). Without compensation for this motion during treatment, the dose coverage to the target may be compromised. However, if compensation of this motion is by an expansion of the margin around the target, a larger PTV margin would result in a larger volume of normal tissues receiving high doses of radiation (7). In order to improve the accuracy of tumor tracking in the prone position and reduce the expansion range of the PTV margin, the XSPT modality was developed primarily for the prone position. XSPT modality combines the spine image registration algorithm with dynamic compensation of respiratory motion. However, relevant reports on XPT technology research are scarce. Although there was only one article quantitatively assessed the dose distribution between supine and sprone position of CyberKnife for spine patiants, the CT of the prone plans were obtained from the CT scanning in the supine position flipped by 180° along the roll axis. So, this experimental scheme does not fully reflect the actual clinical treatment condition. To further determine the actual tracking accuracy of XSPT modality and identify objective criteria for selecting the appropriate tracking modality for the lumbosacral spinal tumor, in our study, the simulation CT scans were acquired in both supine and prone position for each patient with the lumbosacral spinal tumors. In addition, the dosimetric characteristics and setup correction error were compared between the supine and prone positions.

For the dosimetric characteristics, although the DVHs showed that no difference between prone and supine plans was observed in cauda equina D_max_ and D_0.1cc_, the D_1cc_ and D_5cc_ were significantly lower in the prone plans. Meanwhile, the prone plans consistently had a lower dose compared with the supine plans at all dose levels for the bowel. Hence, compared with the supine position, the prone position has a dosimetric advantage. In addition, according to the results, the volume of PTV and intestine in the supine position was larger than that in the prone position. For the PTV, in some patients, the tumor has invaded the paravertebral soft tissue, especially for the tumor near the dorsal muscle, which is deformed by the supine position that compressed the dorsal muscle, so there was some difference in the volume. For the bowel, the shape and position are uncertain due to the peristalsis of the viscera. In addition, the use of the belly board can push the bowel away from the target, so there was uncertainty about the volume of the bowel. However, these volume differences, while statistically significant, are relatively small in clinical evaluation and therefore do not have a large impact on dose distribution.

For the positioning stability, the results showed that the shift deviation in the inferior/superior and anterior/posterior in the prone position was significantly higher than that in the supine position. It indicated that the position deviation of the lumbosacral spine in the direction of anterior/posterior and inferior/superior was not a strict random error but the abdominal movement caused by respiration. The previous study has reported that even in the lumbar spine, respiration-induced target motion in a prone position could not be canceled out completely (13, 14). However, in our study, the average correlation error of synchrony models was less than 1 mm, and the maximum correlation error was less than 1.5 mm. It indicated that the XSPT modality can achieve accurate target tracking in the prone position. On the other hand, using the XSPT modality, the PTV margin can be expanded by 1.5mm to meet the coverage of the target.

In addition, the study found that the fixation devices of patient positioning were also a key factor affecting the stability and repeatability of patient positioning. Several studies had shown that the volume of irradiation to the bowel during pelvic radiotherapy was reduced by using the prone position with a belly board (15, 16). In our study, the results showed that the bowel dose in both low- and high-dose regions in the prone position was consistently lower than that in the supine position. Hence, there was an advantage for the prone position using the prone belly board device over the supine position when the key metric of measuring improvement is the reduction of bowel dose. This result can be explained by the groove design of the prone belly board. The prone belly board could allow the mobile bowel to fall anteriorly and far away from the target, when a patient was positioned in a prone position with the anterior abdomen placed in the groove of the board. It seemed to be more space between the PTV and the bowel, which enabled us to give priority to reducing the radiation dose. In addition, the use of the prone belly board could decrease intra-abdominal pressure made possible by the space afforded. The thermoplastic mesh has a higher fit with the body surface to restrict movement compared with the vacuum cushion (17, 18). Therefore, the prone belly board combined with thermoplastic mesh might decrease the respiratory-induced motion in the prone position. For delivery efficiency, the prone position enables to shorten the effective path length of the photon beam, therefore reducing the number of MU and beams. However, for prone position, the XSPT takes an additional approximately 15 min to establish a synchrony model and therefore has no significant advantage in total treatment time compared with supine position. For most patients with spinal tumors, the prone position offers greater comfort and stability than the supine position. However, for some patients with spinal tumors after surgery cannot be treated in supine position for a long time, only prone position might be used. From our study, we found that the prone position was not superior in terms of comfort, stability, and treatment time. However, our results show that the CyberKnife XSPT model also has significant advantages and can be used for radiotherapy in prone position. In order to better improve the comfort and stability of patient treatment, it is necessary to optimize the positioning fixed equipment. This is also the research that we need to carry out in the future.

## 5 Conclusions

For the patients with lumbosacral spine tumor treated with the CyberKnife, XSPT can correct the errors of prone position caused by respiration in real time and achieve accurate tracking of the tumor. In addition, compared with the supine position, the prone position is able to reduce low and moderate dose irradiation of the intestine and cauda equina and reduce beam numbers and MU.

## Data availability statement

The original contributions presented in the study are included in the article/supplementary material. Further inquiries can be directed to the corresponding authors.

## Ethics statement

Written informed consent was obtained from the individual(s) for the publication of any potentially identifiable images or data included in this article.

## Author contributions

JL, XK and HZ conceived and designed the study. JL, XK, CC and GW performed the experiments. JL wrote the paper. RY and HZ reviewed and edited the manuscript. All authors read and approved the manuscript.
